# Development and Validation of a Machine Learning Model Using Administrative Health Data to Predict Onset of Type 2 Diabetes

**DOI:** 10.1001/jamanetworkopen.2021.11315

**Published:** 2021-05-25

**Authors:** Mathieu Ravaut, Vinyas Harish, Hamed Sadeghi, Kin Kwan Leung, Maksims Volkovs, Kathy Kornas, Tristan Watson, Tomi Poutanen, Laura C. Rosella

**Affiliations:** 1 Layer 6 AI, Toronto, Ontario, Canada; 2 Department of Computer Science, University of Toronto, Toronto, Ontario, Canada; 3 Dalla Lana School of Public Health, University of Toronto, Toronto, Ontario, Canada; 4 Temerty Faculty of Medicine, University of Toronto, Toronto, Ontario, Canada; 5 Temerty Centre for Artificial Intelligence Research and Education in Medicine, University of Toronto, Toronto, Ontario, Canada; 6 Vector Institute, Toronto, Ontario, Canada; 7 Institute of Clinical Evaluative Sciences (ICES), Toronto, Ontario, Canada; 8 Institute for Better Health, Trillium Health Partners, Mississauga, Ontario, Canada

## Abstract

**Importance:**

Systems-level barriers to diabetes care could be improved with population health planning tools that accurately discriminate between high- and low-risk groups to guide investments and targeted interventions.

**Objective:**

To develop and validate a population-level machine learning model for predicting type 2 diabetes 5 years before diabetes onset using administrative health data.

**Design, Setting, and Participants:**

This decision analytical model study used linked administrative health data from the diverse, single-payer health system in Ontario, Canada, between January 1, 2006, and December 31, 2016. A gradient boosting decision tree model was trained on data from 1 657 395 patients, validated on 243 442 patients, and tested on 236 506 patients. Costs associated with each patient were estimated using a validated costing algorithm. Data were analyzed from January 1, 2006, to December 31, 2016.

**Exposures:**

A random sample of 2 137 343 residents of Ontario without type 2 diabetes was obtained at study start time. More than 300 features from data sets capturing demographic information, laboratory measurements, drug benefits, health care system interactions, social determinants of health, and ambulatory care and hospitalization records were compiled over 2-year patient medical histories to generate quarterly predictions.

**Main Outcomes and Measures:**

Discrimination was assessed using the area under the receiver operating characteristic curve statistic, and calibration was assessed visually using calibration plots. Feature contribution was assessed with Shapley values. Costs were estimated in 2020 US dollars.

**Results:**

This study trained a gradient boosting decision tree model on data from 1 657 395 patients (12 900 257 instances; 6 666 662 women [51.7%]). The developed model achieved a test area under the curve of 80.26 (range, 80.21-80.29), demonstrated good calibration, and was robust to sex, immigration status, area-level marginalization with regard to material deprivation and race/ethnicity, and low contact with the health care system. The top 5% of patients predicted as high risk by the model represented 26% of the total annual diabetes cost in Ontario.

**Conclusions and Relevance:**

In this decision analytical model study, a machine learning model approach accurately predicted the incidence of diabetes in the population using routinely collected health administrative data. These results suggest that the model could be used to inform decision-making for population health planning and diabetes prevention.

## Key Points

**Question:**

Can a machine learning model trained on routinely collected administrative health data be used to accurately predict the onset of type 2 diabetes at the population level?

**Findings:**

In this decision analytical model study of 2.1 million residents in Ontario, Canada, a machine learning model was developed with high discrimination, population-level calibration, and calibration across population subgroups.

**Meaning:**

Study results suggest that machine learning and administrative health data can be used to create population health planning tools that accurately discriminate between high- and low-risk groups to guide investments and targeted interventions for diabetes prevention.

## Teaser

This decision analytical model study assesses whether a machine learning model trained on routinely collected administrative health data from a single-payer health system in Canada can be used to predict the onset of type 2 diabetes in the population.

## Introduction

The global incidence and prevalence of diabetes is rising steadily, imposing considerable burden on health care systems. Between 2010 and 2030, it is projected that the prevalence of all forms of diabetes in adults will increase by 69% in developing countries and by 20% in developed countries.^1^ In 2030, it is projected that the prevalence of diabetes will reach 55 million people in the US, 62 million in China, and 87 million in India.^1,2^ Finally, in 2015, the global cost of diabetes was estimated to be $1.31 trillion US dollars.^3^

Serious efforts and investments into the prevention of type 2 diabetes are vital, and it has been well established that prevention programs are effective not only in clinical trials but in pragmatic, real-world settings.^4,5^ However, it has proved difficult to scale diabetes prevention from the individual patient to the population due to systems-level barriers.^6^ These barriers include disparities in socioeconomic status,^7,8,9^ lack of access to healthy foods and medications,^10,11,12^ lack of access to health care,^13,14^ and the built environments in which people at risk of diabetes live.^15,16^ These barriers, many of which are also known as the social determinants of health, contribute to “cascades in care” in which large segments of the population do not meet prevention targets.^17^

Identifying those most in need of interventions (eg, communities that could benefit from subsidies to access healthy foods or diabetes screening and prevention clinics) at the system level by governments, health insurance providers, and public health planners may be hampered by the lack of efficient systems to identify the distribution of risk in the population accurately.^5,18^ Extensive research exists on building diabetes risk prediction models with traditional statistical approaches and machine learning^19,20,21,22,23,24,25,26,27,28,29,30,31,32,33,34^; however, the vast majority of these models are created for direct patient care and not for application at the level of the entire population for public health planning. A systematic review conducted on traditional, statistical diabetes risk scores in 2011 concluded that most risk scores are rarely used because they rely on uncommon tests or were not developed with end users in mind.^34^ The review also concluded that using risk scores on population data sets to identify targets for public health interventions is a promising direction for continued work.^34^ These population-level data sets, also known as administrative health data, are high-dimensional, are impossible to fully explore by clinicians or health system administrators using traditional methods, and represent opportunities for automated, machine learning–based approaches.

We aimed to develop and validate a population-level machine learning model to predict the incidence of type 2 diabetes 5 years before the actual onset of diabetes with high performance using routinely collected administrative health data. The main purpose of our model was to inform population health planning and management for the prevention of diabetes that incorporates health equity. It was not our goal for this model to be applied in the context of individual patient care. We developed and validated our model using a large, contemporary cohort from Ontario, Canada’s single-payer health insurance system that covers all residents. We created our model with the intention that it could be used on data that are routinely collected by governments or health insurance systems, thereby offering efficient, population-level applicability while maintaining robust performance. Our model was assessed for discrimination and calibration, as well as calibration in key demographic subgroups. We also estimated the costs associated with the incident cases predicted by our model each year to demonstrate the financial incentives of using such an approach to target preventive efforts at the health system level.

## Methods

### Study Design and Participants

This decision analytical model study used administrative health services records linked with population and other data holdings covered under a single-payer health system in Ontario, Canada. We used an 11-year period from January 1, 2006, to December 31, 2016. This study obtained ethics approval from the Research Ethics Board at the University of Toronto (protocol No. 37650). The need for informed consent was waived owing to the use of deidentified patient data. In Ontario, all residents are eligible for universal health coverage; therefore, administrative health data cover virtually every resident. Moreover, Ontario is Canada’s most populous province and among the most ethnically diverse populations in the world.^35^ In 2016, it had a population of 13.2 million, of whom almost 30% were immigrants.^35^

The study linked multiple diverse data sources including demographic information, census, physician claims, laboratory results, prescription medication history, hospital and ambulatory usage, and others. Our administrative health data are significantly distinct from electronic medical records. Details on the specific administrative health data that we selected from the Institute of Clinical Evaluative Sciences (ICES) can be found in eTables 1 and 2 in the Supplement.

We randomly sampled 3 000 000 patients linked with Ontario’s Registered Persons Database, with no initial exclusion criteria, which decreased to 2 137 343 after excluding patients not alive as of January 1, 2013, the earliest date of prediction of the model in this study’s design. From this cohort, we also removed patients not residing in Ontario, patients already diagnosed with diabetes, and patients not in contact with the health care system. This last criterion designates patients having a last registered interaction with the health care system before the end of the target window. The proportion of patients in our final cohort with incident diabetes and those without diabetes reflects incidence rates reported in studies at the population level.^36^ In designing and reporting this study, we adhered to the Transparent Reporting of a Multivariable Prediction Model for Individual Prognosis or Diagnosis (TRIPOD) and Strengthening the Reporting of Observational Studies in Epidemiology (STROBE) reporting guidelines.^37,38^

### Model Development

For each patient in the cohort, we partitioned the entire time period into sliding (over the time dimension) patient-time instances that represent a view of the patient at a specific point in time. The detailed diagram of our end-to-end pipeline is shown in eFigure 1 in the Supplement, and further explanations on the instance creation procedure can be found in eMethods 1 in the Supplement. Following a recently proposed approach also training a model at the instance level,^39^ each instance corresponded to pairs made of a 2-year block of the patient’s history and its associated binary diabetes onset label 5 years later. Instances were separated by 3-month gaps, which allowed us to make quarterly predictions.

This sliding-window, multi-instance approach simulated continuous population screening in a practical application and was also conceptually similar to discrete-time survival analysis methods in which covariates are processed in sequential chunks.^40,41,42^ We simulated a system in which the entire cohort was screened every 3 months, and the risk of developing diabetes was computed for each patient. The system’s task was to accurately capture all instances of developed diabetes in the target prediction window (answering the question: Will the patient develop diabetes at any time during the target window?), which required the model to perform well across patients and across time. Three months is a typical update frequency in our administrative health databases; thus, running the model to make a new patient’s future state prediction every 3 months allowed us to constantly refresh the predictions as new data became available.^43^

We partitioned the cohort into 3 nonoverlapping sets of patients, with 1 953 494 patients for model training, 300 000 for validation, and 300 000 for testing. Patients in each set were selected at random. All model developments and parameter selections were performed on the training and validation sets, and the test set was kept untouched for final performance reports. To reduce the time bias, we further partitioned the data in time. For patients in the training set, we used instances that had target windows in the period of January 1, 2013, to December 31, 2014 (2 years, or 8 instances). Similarly, for validation and test sets, only instances with target windows within the periods of January 1, 2015, to December 31, 2015, and January 1, 2016, to December 31, 2016, were used (1 year in each case, or 4 instances). The detailed statistics for each set are summarized in Table 1. Partitioning the training, validation, and test sets in time as well as patients ensured zero overlap between the sets. This process provided a more accurate estimate of performance because, in practice, the model would also be applied to patients who are newly added to the system (ie, unseen during model training and internal validation), and all predictions would be done forward in time compared with the training data.

**Table 1.** Cohort Description^a^

Variable	No. (%)					
	Training (January 2013 to December 2014)		Validation (January to December 2015)		Test (January to December 2016)	
	Total	Positives	Total	Positives	Total	Positives
Full cohort						
Unique patients, No.	1 657 395	23 979	243 442	1874	236 506	1967
Instances, No.	12 900 257	23 979	959 276	1874	927 230	1967
Sex						
Male	6 233 595 (48.3)	12 249 (51.1)	459 715 (47.9)	971 (51.8)	440 433 (47.5)	999 (50.8)
Female	6 666 662 (51.7)	11 730 (48.9)	499 561 (52.1)	903 (48.2)	486 797 (52.5)	968 (49.2)
Age group, y						
<10	1 616 100 (12.5)	205 (0.9)	102 462 (10.7)	14 (0.7)	88 668 (9.6)	8 (0.4)
10-19	1 954 979 (15.2)	358 (1.5)	142 442 (14.8)	32 (1.7)	136 183 (14.7)	32 (1.6)
20-29	1 939 960 (15.0)	696 (4.0)	148 168 (15.4)	75 (4.0)	144 396 (15.6)	79 (4.0)
30-39	1 882 470 (14.6)	2624 (10.9)	140 953 (14.7)	220 (11.7)	135 758 (14.6)	203 (10.3)
40-49	2 108 830 (16.3)	5374 (22.4)	155 409 (16.2)	423 (22.6)	149 244 (16.1)	437 (22.2)
50-59	1 657 299 (12.8)	6353 (26.5)	130 529 (13.6)	486 (25.9)	130 880 (14.1)	524 (26.7)
60-69	987 254 (7.7)	4701 (19.6)	80 069 (8.3)	364 (19.4)	82 448 (8.9)	423 (21.5)
70-79	510 517 (4.0)	2438 (10.2)	39 803 (4.1)	182 (9.7)	40 475 (4.4)	181 (9.2)
80-89	222 638 (1.7)	902 (3.8)	17 637 (1.8)	72 (3.8)	17 239 (1.9)	74 (3.8)
90-100	19 840 (0.2)	53 (0.2)	1761 (0.2)	6 (0.3)	1924 (0.2)	6 (0.3)
Immigration status						
Immigrant	1 537 571 (11.9)	4293 (17.9)	122 532 (12.8)	338 (18.0)	122 607 (13.2)	384 (19.5)
Long-term resident	11 362 686 (88.1)	19 686 (82.1)	836 744 (87.2)	1536 (82.0)	804 623 (86.8)	1583 (80.5)
Race/ethnicity marginalization score, quintile^b^						
1st	19 588 853 (15.2)	3690 (15.4)	144 694 (15.1)	275 (14.7)	136 943 (14.8)	303 (15.4)
2nd	2 083 902 (16.2)	3604 (15.0)	153 306 (16.0)	274 (14.6)	147 340 (15.9)	250 (12.7)
3rd	2 279 478 (17.7)	3711 (15.5)	167 552 (17.5)	304 (16.2)	162 545 (17.5)	318 (16.2)
4th	2 698 267 (20.9)	4441 (18.5)	201 623 (21.0)	355 (18.9)	194 554 (21.0)	366 (18.6)
5th	3 710 695 (28.8)	8126 (33.9)	279 566 (29.1)	642 (34.3)	273 841 (29.5)	703 (35.8)
Deprivation marginalization score, quintile^b^						
1st	3 041 507 (23.6)	4339 (18.1)	227 873 (23.8)	366 (19.5)	220 439 (23.8)	358 (18.2)
2nd	2 566 726 (19.9)	4569 (19.1)	190 232 (19.8)	333 (17.8)	185 106 (20.0)	383 (19.5)
3rd	2 442 622 (18.9)	4572 (19.1)	182 185 (19.0)	359 (19.2)	173 694 (18.7)	372 (18.9)
4th	2 288 370 (17.7)	4714 (19.7)	170 096 (17.7)	394 (21.0)	164 405 (17.7)	420 (21.4)
5th	2 391 970 (18.5)	5378 (22.4)	176 355 (18.4)	398 (21.2)	171 579 (18.5)	407 (20.7)

^a^ We give the number of patients, number of instances, and associated number of positive data points for the training, validation, and test sets. Note that the number of positive patients and instances match exactly as a patient can only be diagnosed once with diabetes; we also give the distribution of each set in terms of sex, age, and immigration status.

^b^ Race/ethnicity and deprivation marginalization scores quantify the degree of marginalization within each dissemination area according to ethnic concentration and material deprivation. A dissemination area typically encompasses a few hundred inhabitants. These 2 scores are quintiles ranging from 1 to 5 based on each patient's history from the 2004-2008 period, where 5 represents a highest degree of marginalization.

We examined more than 300 features derived from demographic details, geographic information, chronic conditions, and health care use history. Stationary demographic features included sex, birth year, immigrant status, and country of origin. Geographic information comprised residence statistics and measures of area-level socioeconomic status from recent census surveys at the Dissemination Area (400-700 individuals) level. Race/ethnicity and material deprivation marginalization scores were built with the Ontario Marginalization Index and reflected neighborhood-level socioeconomic information.^44^ Health care use included information on physician or specialist visits, emergency department visits, laboratory results, hospitalizations and ambulatory usage, and prescription history during the observation window (eFigure 2 and 3 in the Supplement). Extensive details on feature engineering can be found in eMethods 2 in the Supplement.

We trained the gradient boosting decision tree model implemented in Python in the XGBoost (The XGBoost Contributors) open source library.^45^ The gradient boosting decision tree model was chosen owing to its ability to handle different feature types and missing values and good support for explainability. Not all patients have values for all features, given variation in health care use and laboratory tests. We did not remove patients with missing values, because XGBoost can still produce predictions without complete case data. Details on the XGBoost model parameters can be found in eMethods 3 in the Supplement. Results for different buffer sizes can be found in eTable 3 in the Supplement. XGBoost was compared with logistic regression in eTable 4 in the Supplement.

### Statistical Analysis

**Figure 1.**
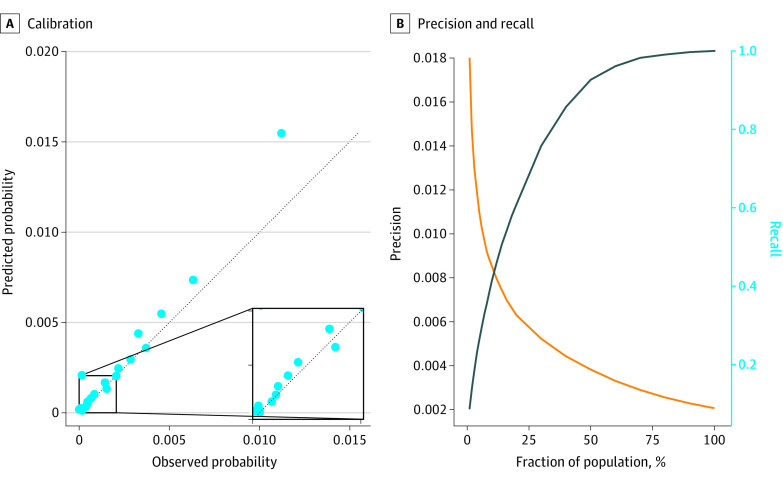
Diabetes Onset Prediction Performance A, Calibration is assessed visually with a calibration curve composed of 20 population bins of equal size. B, Precision and recall curves are displayed. The left y-axis corresponds to precision, and the right y-axis to recall. The test area under the receiver operating curve is 80.26.

To assess model performance, given the extremely unbalanced class ratio, we tracked the area under the receiver operating characteristic curve (AUC). The AUC is commonly used for such prediction tasks and is robust to class imbalances.^46^ We reported the model’s calibration curve in Figure 1 for a visual verification of calibration. For practical application, it was relevant to focus on high-risk patients (ie, those with the highest predicted probability of developing type 2 diabetes) given that our cohort is at the population level. To evaluate the model’s performance on the highest-risk patients, we display the precision and recall curves in eFigure 4 in the Supplement.

**Figure 2.**
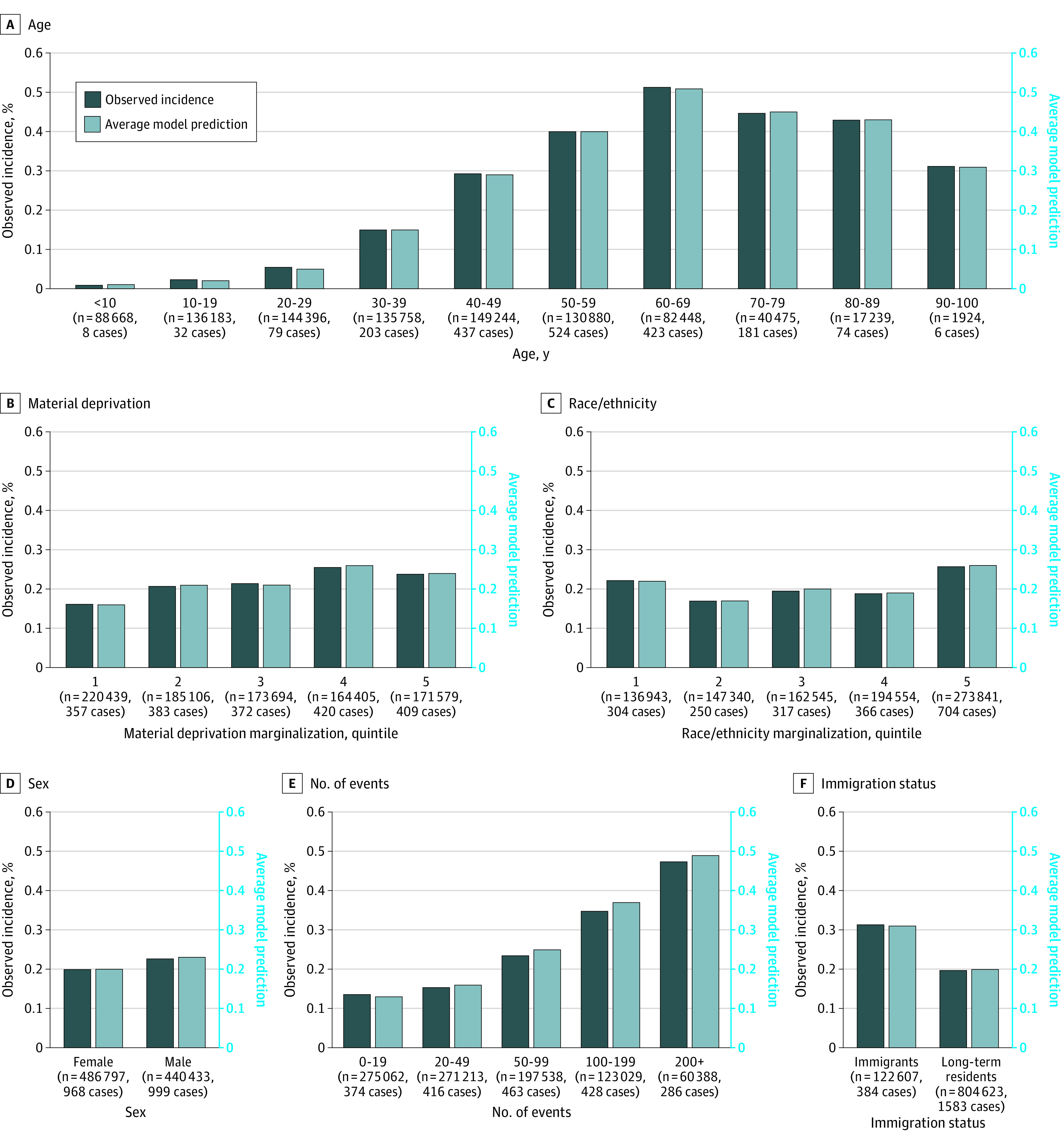
Diabetes Onset Calibration Across Population Groups The model is evaluated on specific subsets of the population: sex (2 categories), age (10 bins of 10 years), immigration status (2 categories), race/ethnicity marginalization score (5 quintiles), material deprivation marginalization score (5 quintiles), and number of events in the observation window (5 categories). We display the incidence rate (left y-axis, dark blue bars), average model prediction (right y-axis, light blue bars), and number of positive cases within each subset. The size of each subset can be read on the x-axis. Note that incidence rates can vary dramatically between subsets, especially for age, making comparisons between subsets challenging.

As shown in Figure 2, we evaluated the model on several subsets of the data, separating patients by sex, age, immigration status, marginalization (in terms of both race/ethnicity and material deprivation), and number of events. The number of events was defined as the total number of times that a patient interacted with the health care system in any way during the observation window. It was possible for the patient to have zero events during 1 or several observation windows, in which case the only nonzero variables in the patient’s instance features would be never-missing stationary variables, such as the country of birth or sex. We reported the feature contribution using the Shapley values, further described in eFigure 3 in the Supplement.^47,48^

To assess the financial burden of the cohort of patients with diabetes, we used a costing algorithm developed by ICES.^49^ This algorithm provides the total public health care expenditure per year for each patient, based on the patient’s billing information across health care services. With this costing algorithm, we derived the annual cost of the cohort of patients with diabetes in Ontario, as well as the annual change in this cost. This cohort grows over time as the number of patients newly diagnosed with diabetes is greater than the number of patients with diabetes who die each year. We used this algorithm in combination with our model’s predictions to estimate how cost-effective the policies implemented with the model could be. This process was done by sorting patients in the test set by decreasing model prediction (from the highest likelihood of getting diabetes as predicted by the model to the lowest) and computed the cumulated cost of these patients. All costs were reported in 2020 US dollars. Data were analyzed using SAS Enterprise software, version 6.1 (SAS Institute Inc) from January 1, 2006, to December 31, 2016.

## Results

After applying the exclusion criteria, the resulting cohort sizes were 1 657 395 patients for training (12 900 257 instances; 6 666 662 women [51.7%]), 243 442 for validation, and 236 506 for testing (Table 1). That is, we used 83.7% of the patients in our analytic cohort, substantially more than similar studies.^23^ A total of 416 151 patients were excluded: 191 999 patients owing to date of last contact being before the earliest possible target window of their set (training, validation, or test), 103 613 because they were immigrants who arrived in Canada after the end of their observation window, and 120 539 because they were already diagnosed with diabetes. The training, validation, and test sets contained 12 900 257, 959 276, and 927 230 patient instances, respectively. All of the results reported in this section are referring to the test set unless mentioned otherwise.

Figure 1 displays the performance of the model in different evaluation setups. We computed the AUC for all instances in the test set, spanning all 4 quarters of 2016 for each test patient. As seen in Figure 1, the model achieved a test AUC of 80.26 (range, 80.21-80.29) on this held-out set. The calibration curve contained 20 bins with an equal number of patients and was well aligned with the identity line, which showed a good calibration overall. The only exception was the last bin, which showed model overprediction for high-risk patients.

As shown in Figure 2, we evaluated the model on several partitions of the test population, and for each subset, we reported the size, incidence rate, and average model prediction. Incidence rates varied significantly across subsets: it was less than 0.1% for patients aged 20 to 29 years, whereas it was greater than 0.5% among those aged 60 to 69 years. We used the following partitions: sex, age, immigration status, material deprivation marginalization, race/ethnicity marginalization, and number of events in the observation window. We observed that, for all partitions, the model was well calibrated across all subsets except for the number of events; for a higher number of events, the model was slightly overpredicting.

We included an analysis that demonstrates how such a prediction model could be informative at the population level by examining predicted risk across the population into groups that can be segmented for health system planning, such as targeted interventions or resources. Table 2 depicts an analysis of the model prediction bins and the same analysis within subgroups of the population. Given the incidence rate of 0.2%, the top 1% constituted the high-risk patients, the next 5% were moderate-risk patients, the next 15% were low-risk patients, and the remaining 79% were negligible-risk patients. Analysis of these risk bins reflected the variables and thresholds used by the model to make predictions.

**Table 2.** Model Prediction Risk Levels^a^

Bin	Age, mean	Individuals, %		Time in Canada, y	Marginalization scores		HbA_1c_, mean
		Women	Immigrants		Ethnicity	Deprivation	
Model prediction							
Top 1%	58.3	59.6	38.8	17.3	4.22	3.63	5.84
Next 5%	59.4	42.3	26.5	18.4	3.85	3.45	5.81
Next 15%	58.3	40.8	16.5	19.4	3.44	3.15	5.73
Bottom 79%	31.8	55.3	11.4	19.7	3.38	2.87	5.53
Label							
Positive	53.7	49.2	19.5	19.1	3.54	3.15	5.92
Negative	37.4	52.5	13.2	19.6	3.42	2.95	5.63

Abbreviation: HbA_1c_, hemoglobin A_1c_.

^a^ In the first setup, we rank patients by their model's output in decreasing order, then bin them into 4 categories: top 1%, next 5% (between top 1% and top 6%), next 15% (between top 6% and top 21%), and the remaining 79%. For each bin, we display statistics pertaining to general demographic factors (mean age, fraction of women, fraction of immigrants and time in Canada for immigrants) and socioeconomic factors (race/ethnicity and deprivation marginalization scores of the neighborhood), as well as the mean HbA_1c_. Means are computed across nonmissing values from patients within each bin. For instance, time in Canada is computed only for immigrants of each model output bin as the value is missing for long-term residents. The second setup evaluates the same variables but when splitting patients according to their label (positive or negative).

Patients developing diabetes were typically much older (mean age, 53.7 years) than patients who did not develop diabetes (mean age, 37.4 years) within the time frame of our study. Similarly, the very high-risk patients selected by our model had a mean age of 58.3 years. The model evaluated a greater proportion of immigrants considered to be at high risk. Patients at higher risk were more likely to live in neighborhoods with a high concentration of ethnic minority groups and material deprivation, as 4.22 and 3.63 were the mean scores, respectively, for high-risk patients compared with 3.38 and 2.87 for low-risk patients.

**Figure 3.**
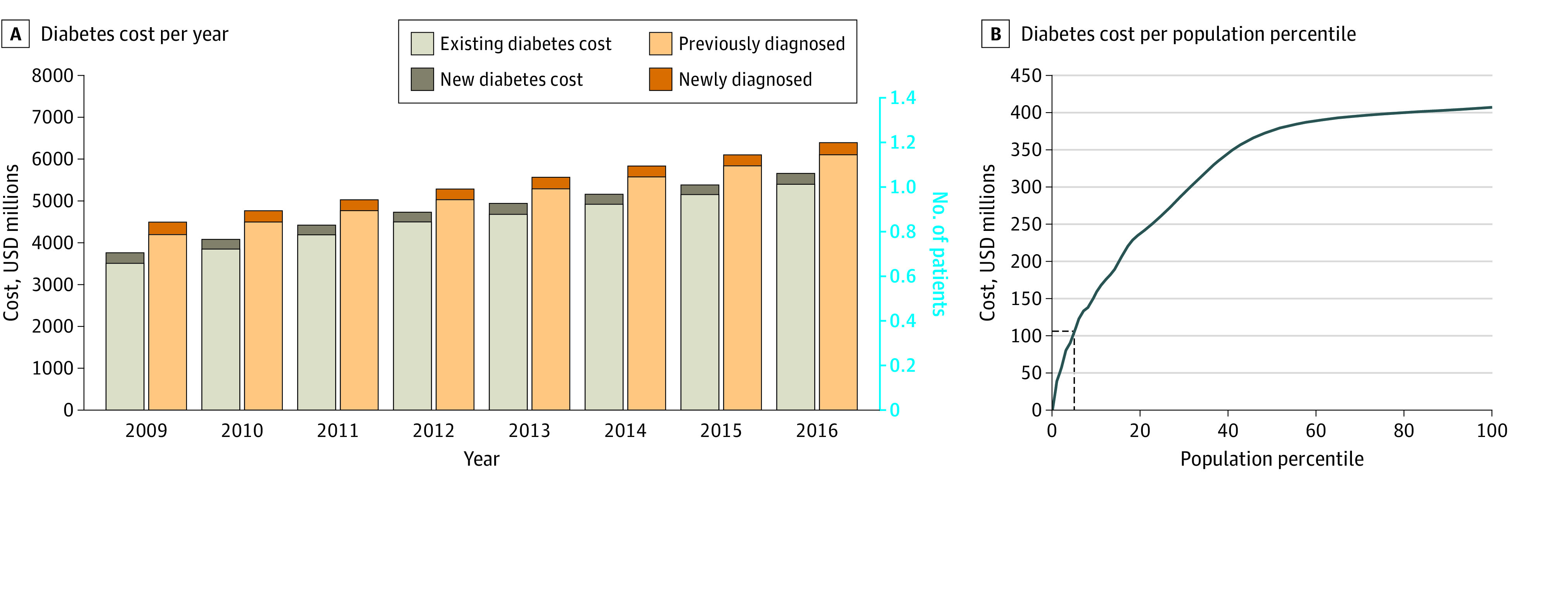
Estimation of the Total Cost of the Cohort Predicted to Develop Diabetes in Ontario from 2009-2016 Diabetes cost per year (A) and per population percentile (B) are displayed. The 5% most at-risk patients concentrate 26% of the total cost. USD indicates US dollars.

In Figure 3, we display an estimation of the total cost of the cohort predicted to develop diabetes in Ontario from 2009 to 2016. Figure 3A represents an estimation of this cohort and the associated cost after scaling our cohort to the entire population of Ontario. Although the number of patients with diabetes is estimated to be 785 000 with an associated cost of $3.5 billion in 2009, these figures increased to 1 144 000 and $5.4 billion, respectively, only 7 years later. The cohort with diabetes grew at an average of 51 800 new patients per year between 2009 and 2016, which added, on average, $242 million per year to the financial burden of diabetes. Moreover, in Figure 3B, the patients who were predicted to be at the highest risk by our model composed a large fraction of the cost: moderate-risk and high-risk patients were 5% of the population but represented 26% of the total diabetes cost.

Further results on the performance of our model are displayed in eFigures 2, 3, and 4 and eTable 3 in the Supplement. We conducted an ablation study over data sources, analyzed feature contribution from each data set, and reported precision and recall curves and AUC results for buffers of 1 year and 3 years, respectively.

## Discussion

This decision analytical model study found that accurate prediction of type 2 diabetes onset at the population level 5 years in advance was possible solely from routinely collected administrative health data for the purposes of public health planning and health resource allocation. It was not our goal for this model to be applied in the context of individual patient care. Our model was trained and validated on more than 2 million patients, which, to our knowledge, is one of the largest cohorts for predicting diabetes incidence. Our model showed consistent calibration across sex, immigration status, racial/ethnic and material deprivation, and a low to moderate number of events in the health care history of the patient. The cohort was representative of the whole population of Ontario, which is itself among the most diverse in the world.^50^ The model was well calibrated, and its discrimination, although with a slightly different end goal, was competitive with results reported in the literature for other machine learning–based studies that used more granular clinical data from electronic medical records without any modifications to the original test set distribution.^23,24,25^

Assessing risk in populations is the basis of health system planning and a critical element of diabetes prevention.^51,52^ When managing risk in populations, there are critical questions regarding the most efficient usage of resources, and without a comprehensive estimate of risk in populations, strategies can be costly and ineffective. Furthermore, it is widely recognized that the prevention of diabetes is not only influenced by factors at the individual level but must be complemented by whole population approaches, such as food policies and environmental changes.^6^ The use of machine learning methods for predicting risk in populations offers an important opportunity to inform resource and policy-level decisions that can change diabetes risk trajectories as well as allow for more efficient targeting of resources within a health system.

The growing burden of diabetes is a challenge faced by other jurisdictions across the globe.^1,2,3^ Continuous risk assessment using the multi-instance approach we proposed could reduce this cost through the targeting of preventive health measures, even more so given the fact that our model did not require additional data collection. Such an approach could be feasible in countries such as the UK, Australia, New Zealand, and the Scandinavian countries, which have large, administrative databases suitable for linkage.^53,54,55,56,57^ Furthermore, this approach could also be deployed in populations covered under a singular health insurance system, such as Medicare or private insurers.^58^

Our features not only captured each patient’s medical history but also included the social and demographic determinants of health, which are important predictors of a patient’s overall risk of developing diabetes and are often missing in clinical data sources.^59,60,61^ Moreover, the calibration of our machine learning model across demographic subgroups suggests that it may be possible to apply it to target-specific population segments with preventive measures (Table 2 and Figure 3). Diabetes prevention strategies can be targeted toward those above a certain risk threshold.^62^ Our model results suggest that older patients from the most marginalized neighborhoods in terms of race/ethnicity and material deprivation were at the highest risk and may therefore benefit the most from preventive measures. Given the growing costs associated with the diabetes cohort, our work suggests a quantitative financial incentive toward the direction of preventive measures that consider those at greatest risk, including from a socioeconomic perspective.^59^ Because our machine learning model included social determinants of health that are known to contribute to diabetes risk, our population-wide approach to risk assessment may represent a tool for addressing health disparities.^59,63,64^

### Strengths and Limitations

Our study approach had several strengths. Owing to the nature of administrative data, such an approach could be applied to other chronic diseases. In 2009, 24.3% of Ontarians were found to be affected by multiple comorbidities.^65^ Accurately forecasting other prominent chronic conditions, such as hypertension, could lead to potential considerable reductions in health care costs while also improving the health and well-being of the population. Similar work to create risk prediction models has been done in a primary prevention cohort from New Zealand to determine 5-year cardiovascular disease risk, and research from the UK reinforces that reducing cardiovascular event risk by even 1% would result in both large cost savings and improved population health.^54,66^ Moreover, we included a detailed calibration assessment, both overall and within key population subgroups, which suggests that our model did not only have strong discrimination but was well calibrated in a diverse population.^67^ Finally, the choice of using a gradient boosting machine model permitted the usage of Shapley values to enhance explainability.^68^ Our proposed approach also had some important limitations. First, there was the potential for misclassification of patients with type 1 diabetes given limitations with the algorithm used in label construction of type 1 and type 2 diabetes.^69,70^ Of the roughly 2% to 3% of individuals aged 20 years or younger who tested positive for diabetes, we are uncertain how many were actually diagnosed with type 1 diabetes. However, we chose not to exclude younger patients in our cohort owing to the rising incidence of type 2 diabetes in youths and young adults.^71,72^ Second, the input administrative health data were highly heterogeneous: only 23.4% of patients had at least 1 laboratory value, and only the patients older than 65 years had a prescription history. We believe that more consistency and fewer missing values in the input data would improve the model’s discrimination. Third, administrative data often does not capture certain features known to be highly predictive of diabetes onset, such as body mass index; however, we achieved competitive performance when our machine learning model was compared to those trained on richer sources of data while allowing for applicability at the population level. Fourth, although we can interpret the model’s decisions and the way it splits variables to separate patients into risk score categories, the model strictly captured correlations in the data and not causal pathways. Finally, our model would need to be further validated through prospective studies before deployment.

## Conclusions

In this decision analytical model study, we developed and validated a population-level machine learning model to predict the incidence of type 2 diabetes 5 years ahead in a large, contemporary cohort from Ontario, Canada’s single-payer health system. Study results suggest that our model had strong discrimination and was robust in calibration across several subgroups, including sex, immigration status, race/ethnicity marginalization, and material deprivation marginalization. Following external and prospective validation, our findings suggest that administrative health data and machine learning may be leveraged for the continuous risk assessment and cost-effective targeting of prevention efforts of type 2 diabetes at the population level with a focus on health equity.

## Notes

Supplement. **eFigure 1.** Overview of Our Approach **eMethods 1.** Instance Creation Process **eTable 1.** Comparing Electronic Medical Records vs Administrative Health Data **eTable 2.** Descriptions of Administrative Health Datasets Used **eMethods 2.** Feature Engineering **eMethods 3.** Model Development and Evaluation **eFigure 2.** Ablation Study **eFigure 3.** Feature Contribution **eFigure 4.** Model Precision and Recall Curves **eTable 3.** Results With Other Buffer Sizes **eTable 4.** Comparison Against Logistic Regression Baseline eReferences
