# Supplementary material for: Development and Validation of a Machine Learning Model Using Administrative Health Data to Predict Onset of Type 2 Diabetes
Source: JAMA Netw Open. 2021 May 25;4(5):e2111315. doi: 10.1001/jamanetworkopen.2021.11315 (PMC8150694; doi:10.1001/jamanetworkopen.2021.11315)
Supplement: Supplement. — eFigure 1. Overview of Our Approach eMethods 1. Instance Creation Process eTable 1. Comparing Electronic Medical Records vs Administrative Health Data eTable 2. Descriptions of Administrative Health Datasets Used eMethods 2. Feature Engineering eMethods 3. Model Development and Evaluation eFigure 2. Ablation Study eFigure 3. Feature Contribution eFigure 4. Model Precision and Recall Curves eTable 3. Results With Other Buffer Sizes eTable 4. Comparison Against Logistic Regression Baseline eReferences [file jamanetwopen-e2111315-s001.pdf]

Supplementary Online Content

Ravaut M, Harish V, Sadeghi H, et al. Development and validation of a machine learning model using administrative health data to predict onset of type 2 diabetes. *JAMA Netw Open*. 2021;4(5):e2111315. doi:10.1001/jamanetworkopen.2021.11315

eFigure 1. Overview of Our Approach

eMethods 1. Instance Creation Process

eTable 1. Comparing Electronic Medical Records vs Administrative Health Data

eTable 2. Descriptions of Administrative Health Datasets Used

eMethods 2. Feature Engineering

eMethods 3. Model Development and Evaluation

eFigure 2. Ablation Study

eFigure 3. Feature Contribution

eFigure 4. Model Precision and Recall Curves

eTable 3. Results With Other Buffer Sizes

eTable 4. Comparison Against Logistic Regression Baseline

eReferences

This supplementary material has been provided by the authors to give readers additional information about their work.

© 2021 Ravaut M et al. *JAMA Network Open.*

eFigure 1. Overview of our Approach

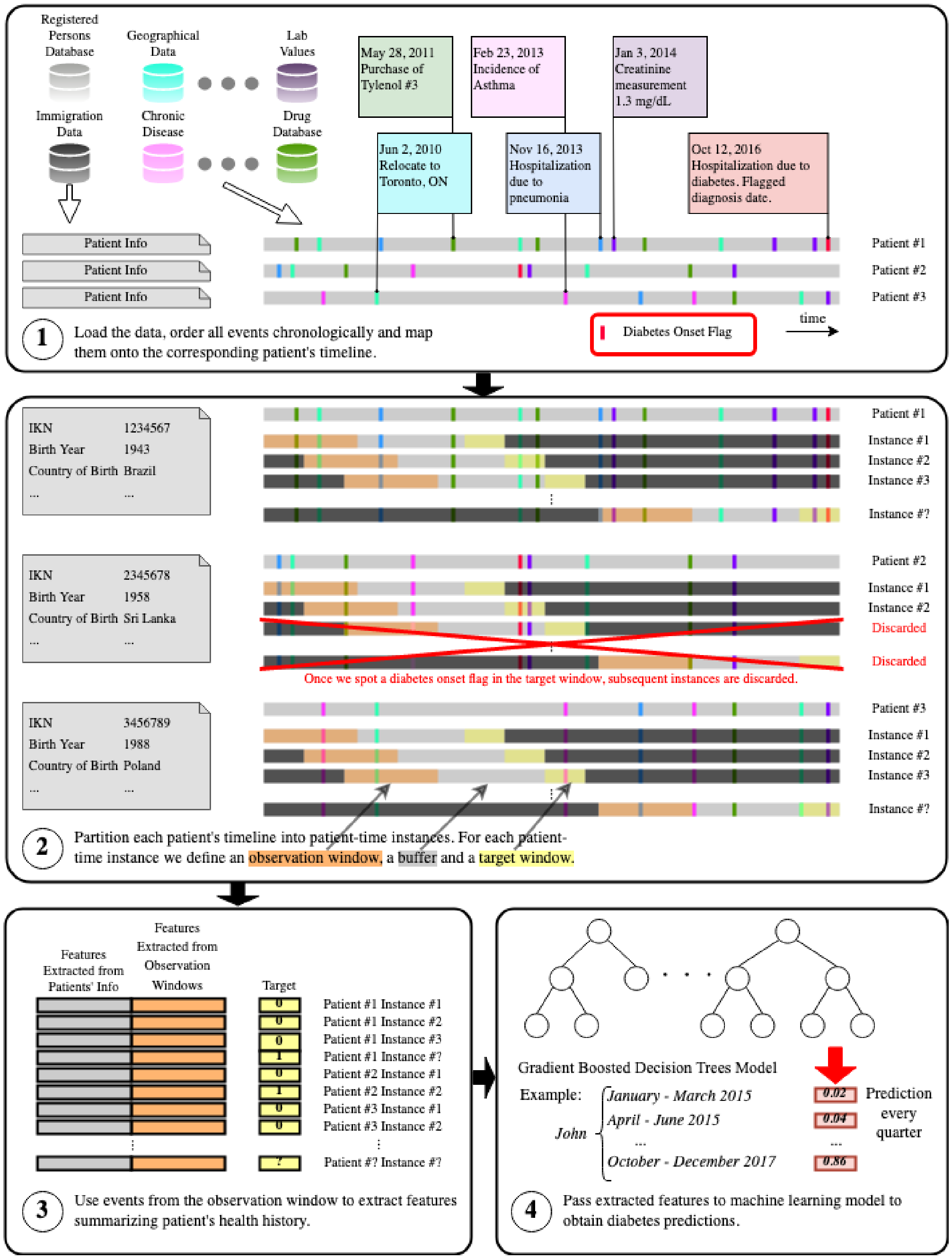

© 2021 Ravaut M et al. *JAMA Network Open.*

eMethods. Instance Creation Process

The data was accessed at ICES, which is an independent, non-profit research institute, whose legal status under Ontario's health information privacy law allows it to collect and analyze healthcare and demographic data, without consent, for health system evaluation and improvement. The diverse data sources from ICES were linked using the unique encoded identifiers from the Registered Persons Database (RPDB), a central registry from the province's universal single-payer healthcare system. This identifier enables linkage across datasets, as depicted in eFigure 1, and contains basic demographic information, including sex, age and geographical residence information that we used in our model.

eTable 1 offers a comparison between the two types of AHD data and EMR. The study by Razavian *et al.* is the closest to ours as it also leverages administrative health data [1]. We chose two recently published studies applying machine learning to diabetes as examples of studies using EMR data [2,3]. EMR data typically are more complete on laboratory values, and sometimes are restricted to include only patients with non-missing values. They may also contain family history or lifestyle variables. AHD are characterized by an abundance of claims and the associated diagnosis codes. They also usually contain drug history and laboratory values, but each data source is typically very sparse, with very few variables that are never missing across patients. AHD may also include geographical or socioeconomic variables, as is the case with our study. While setting a system collecting and building EMRs can be a years-long process [4], AHD forms the first layer of healthcare data, and is automatically collected in single-payer healthcare systems and health insurance systems.

For each patient, we extracted data on healthcare utilization and services accessed from the following sources: physician and emergency claims from the Ontario Health Insurance Plan (OHIP), hospitalization history from the Discharge Abstract Database (DAD), emergency services from the National Ambulatory Care Reporting System (NACRS) and prescription medication claims for individuals aged 65 years or above and those receiving social assistance. Diabetes-related laboratory test results were obtained from the Ontario Laboratory Information System (OLIS). The Ontario portion of the Immigration, Refugees and Citizenship Canada (IRCC) database was used to identify immigration status and country of birth. Neighbourhood-level measures of socioeconomic status were obtained with the 2001, 2006 and 2011 Canadian censuses. Finally, patient deaths that occurred during the observation period were identified from the Office of Registrar General-Deaths (ORG-D) database. A detailed description of all the data sources can be found in Table S2.

The health event data from the observation window was used to extract features that summarize a patient's history at that point in time. We found that the two-year window was sufficient to obtain the necessary information on the predictor features. As shown in eFigure 1, the extracted features were then fed into the model and instance-level diabetes onset predictions were generated for the target window. Despite leading to a lower incidence rate compared to large values, we found that a three-month period had greater predictive performance than a larger window of six months or one year. Furthermore, a three-month target window is designed for the generation of acute and continuous predictions every three months from the patient's health history, giving granularity on the patient's monitoring. If diabetes onset happens after the end of the current target window, the target window of the following instances would capture it.

To determine the 5-year diabetes onset label for a given patient, we used the Hux algorithm in its 2016 version. [6] The Hux algorithm is a validated method used to build the Ontario Diabetes Dataset (ODD) registry, reaching a sensitivity of 86% and a specificity of 97%, when compared to physician labels. We augmented this algorithm with a criteria on the HbA1c values. That is, the earliest date a patient had an HbA1c reading greater than or equal to 6.5 (when such reading is available on the patient) was set to be the onset date. When both HbA1c and HUX onset dates existed, we determined the onset date to be the earlier of the two. If a target window contains diabetes incidence, all subsequent target windows are discarded as irrelevant since the patient has already been diagnosed.

© 2021 Ravaut M et al. *JAMA Network Open.*

eTable 1. Comparing Electronic Medical Records vs Administrative Health Data

**Study type**	**EMR**	**EMR**	**AHD**	**AHD**
**Study**	Cahn (2020)	Choi (2019)	Razavian (2015)	Ours (2020)
Genetic information	No	No	No	No
Family history	No	Yes	No	No
Lifestyle, health surveys	No	Yes	No	No
Ethnicity	No	Yes	No	No, only marg.
Socio-economic factors	No	Yes	No	Yes
Geography	No	No	No	Yes
Comorbidities	No	Yes	Yes	Yes
Drug history	Yes	Yes	Yes	Yes, partially*
Diagnosis history	No	No	Yes	Yes
BMI or weight	Yes	Yes	Yes, partially	No
HbA1c	Yes	Yes	Yes, partially	Yes, partially**
Glucose	Yes	Yes	Yes, partially	Yes, partially
Triglycerides	Yes	Yes	Yes, partially	Yes, partially

Administrative Health Data (AHD). Typically, AHD lacks the presence or coverage over all patients for key variables, especially among laboratory values.

* 17.5% of patients in our cohort have drug history.

** 23.4% of patients have at least one lab value, and 16.1% have at least one hbA1c value

eTable 2. Descriptions of Administrative Health Datasets Used

Dataset	Description
Registered Persons Database (RPDB)	Provides basic information about anyone who have ever received an Ontario health card number. Key data variables include date of birth, sex, geographical information, and time periods for which an individual was eligible for coverage under the Ontario Health Insurance Plan (OHIP). All health card numbers are encoded before being linked to other databases at ICES.
IRCC Permanent Residents Database (CIC)	Contains records for over three million individuals at the time of landing in Ontario from January 1985 to May 2017, and is linked to the RPDB with a 86.4% overall linkage rate. Data include permanent residents' demographic information such as country of birth and landing date.
Ontario Census Area Profile (CENSUS)	Self-reported information collected during population census in 2001 and 2006.
Ontario Marginalization Index (ONMARG)	Socio-economic neighborhood information collected in 2001 and 2006 census.
Postal Code Conversion File (PCCF)	Geographical information such as latitude or longitude.
Local Health Integration Network (LHIN)	Describe which LHIN does the patient refer to. Ontario is partitioned into 14 different LHINs
REF	Further stationary data such as sex.
Ontario Health Insurance Plan (OHIP)	The OHIP claims database contains information on inpatient and outpatient services provided to Ontario residents eligible for the province's publicly funded health insurance system by fee-for-service health care practitioners (primarily physicians) and shadow billings for those paid through non-fee-for-service payment plans. The main data elements include patient and physician identifiers (encrypted), code for service provided, date of service, associated diagnosis, and fee paid. We also extracted OHIP emergency claims data using OHIP Emergency Services (ERCLAIM) dataset, which uses a macro to extract emergency claims data from OHIP claims (one record per emergency service).

Ontario Drugs Benefit Claims (ODB)	The ODB database contains prescription medication claims for those covered under the provincial drug program, mainly: those aged 65 years and older, nursing home residents, patients receiving services under the Ontario Home Care program, those receiving social assistance, and residents eligible for specialized drug programs. Main data elements include drug identifier, quantity, number of days supplied, date dispensed, cost, and patient, pharmacy and physician identifiers.
Discharge Abstract Database (DAD)	The DAD is compiled by the Canadian Institute for Health Information and contains administrative, clinical (diagnoses and procedures/interventions) and demographic information for all admissions to acute care hospitals, rehabilitation, chronic, and day surgery institutions in Ontario. At ICES, consecutive DAD records are linked together to form episodes of care among the hospitals to which patients have been transferred after their initial admission.
National Ambulatory Care Reporting System (NACRS)	The NACRS is compiled by the Canadian Institute for Health Information and contains administrative, clinical (diagnoses and procedures), demographic, and administrative information for all patient visits made to hospital- and community-based ambulatory care centers (emergency departments, day surgery units, hemodialysis units, and cancer care clinics). At ICES, NACRS records are linked with other data sources (DAD, etc.) to identify transitions to other care settings, such as inpatient acute care or psychiatric care.
Ontario Laboratory Information System (OLIS)	A system that connects hospitals, community laboratories, public health laboratories and practitioners to facilitate the secure electronic exchange of laboratory test orders and results. This database provides results for routine laboratory tests, including HbA1c, lipids, serum, creatine, and albumin/creatine ratio from 2006 onwards.
OHIP Emergency Claims Dataset (ERCLAIM)	More specific details for emergency claims.

Ontario Diabetes Database (ODD)	We identified patients living with diabetes from a validated ICES derived registry of all Ontarians identified as having diabetes (prevalent cases) since 1991, which demonstrated sensitivity of 86% and a specificity of 97%. An individual is flagged with diabetes (and included in ODD) if one of the following conditions is met: (1) Two OHIP (physician) claims with diabetic diagnostic code within a two-year period; (2) One hospitalization (in DAD) with diabetic diagnostic code; or (3) A single OHIP claim with a fee code for diabetes management, insulin therapy support, diabetes management assessment. Such code belongs to the following list: Q040, K029, K030, K045 and K046. In fact, we used the 2016 version of ODD, where the algorithm is updated to what we described here. It applies to all individuals in RPDB (even before 2016)}. The diabetes diagnostic codes are given by ICD-9 250.xx or ICD-10 E10-E14
ASTHMA, CHF, HYPER, OCCC, OMID, ORAD	Derived cohorts for the six respective chronic diseases: asthma, congestive heart failure, hypertension, Crohn's disease, myocardial infarction and rheumatoid arthritis. These datasets contain yearly binary flags for both prevalence and incidence of the associated disease for the patient.

We list all the datasets used as input features in our model and their role in the administrative health data at ICES.

eMethods 2. Feature Engineering

We did not perform any pre-processing of continuous variables, except for laboratory results. Laboratory results can be reported in different units, such as mg/L and g/L, and we standardized the unit before doing feature extraction. One-hot encoding was used for all categorical variables, and we discarded categories that appeared with a frequency of less than 1%. Removing infrequent categories significantly reduced the feature size and improved model generalization. As reported in previous studies, we also found that events in the observation window occur in highly irregular patterns. [7] Patients would typically have clusters of activity (multiple doctor/ER visits, laboratory tests, etc.) followed by quiet periods with few events. To summarize these patterns we performed various aggregations over different time intervals within the two year observation window. For time aggregation we counted events in the last month, quarter, 6 months, year, etc.

For event aggregation we combined events of the same type such as doctor visits by physician specialty and prescription medication by drug type. This double aggregation resulted in features such as “number of ophthalmologist visits in the last month" and “total quantity of drug X prescribed in the last year". We found such features to be highly informative for onset prediction.

During feature selection we adopted a greedy approach, and computed multiple combinations of time and event aggregation. These features were then incrementally added into the model and retained only if the validation set performance improved. Note that throughout this process, to prevent any model bias, the test set remained untouched and was only used for the test performance computation of the final model.

In addition to event aggregation, we included other features that summarize a patient's recent medical history. To estimate the recurrence frequency we computed time between consecutive events, as well as time since the most recent event. The goal was to estimate whether certain events are becoming more frequent or occur with a specific time pattern.

Moreover, we compared each patient’s event history with histories from patients in the same sex, age and immigration status groups. Within-group comparisons can identify “outlier" patients whose progression of condition trajectory deviates significantly from other patients. [8]

All feature selection here was performed in a similarly greedy fashion by incrementally adding subsets of features to the model. After multiple rounds of feature selection we obtained a set of approximately 300 features that maximized the validation AUC, and used this set for all further experiments.

© 2021 Ravaut M et al. *JAMA Network Open.*

eMethods 3. Model Development and Evaluation

To find optimal hyperparameters we performed grid search by first specifying ranges for each hyper parameter, and then exhaustively evaluating on points selected from those ranges. After grid search we selected the following settings: a tree depth of 10, learning rate of 0.05, minimum child weight of 50, α= 0.3, γ= 0.1, λ= 0.5, column sample by tree of 0.8 and column sample by level of 0.9 (relevant XGBoost parameter documentation can be found here: https://xgboost.readthedocs.io/en/latest/parameter.html). Since incidence rate for onset is typically lower than 1%, we under-sampled negative instances by a factor of up to 20x to balance the training data. [9,10,11] After training, the output probabilities from the model were re-calibrated using the approach proposed by Dal Pozzolo *et al.* [12]

We reported the feature contribution with the Shapley values (see eFigure 3). [13,14] Specifically, after the model was trained, to estimate the contribution for each type of feature, we averaged absolute Shapley values over a sample of 10,000 instances selected at random from the test cohort. These feature contributions vary from an instance to another, and we found that 10,000 was a sample large enough to get stable Shapley values.

© 2021 Ravaut M et al. *JAMA Network Open.*

eFigure 2. Ablation Study

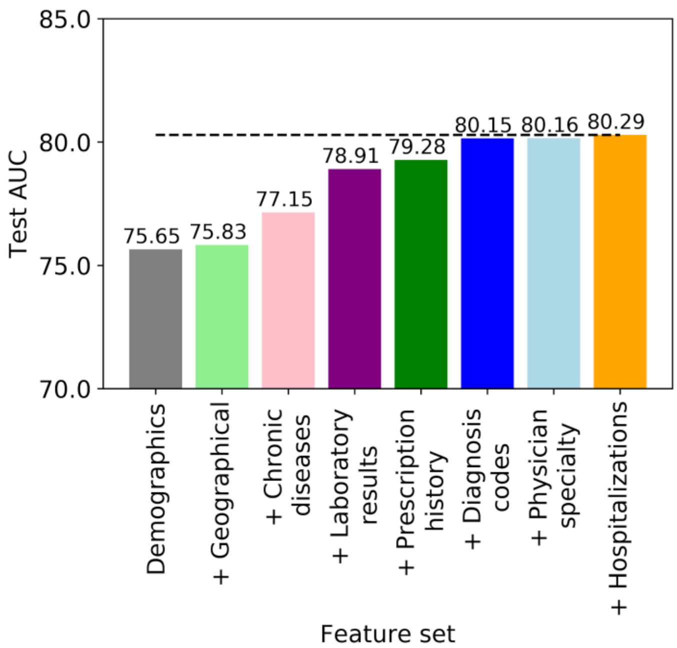

eFigure 2 shows an ablation study of the model performance under different training setups. We sequentially re-train the model with an increasing number of variables. We first train with only the demographics features, then add the ones corresponding to geographical information and re-train, then repeat the process for all types of input data until covering all variables. From this incremental process, we see that different types of data contribute differently to the model's performance. Chronic diseases (+1.32 AUC), laboratory values (+1.75 AUC) and diagnosis codes (+0.87 AUC) give substantial AUC gains, while geographical information, prescription history, physician specialty and hospitalizations contribute marginally. It is important here to note that the absolute gains from each data type depend on the order in which the data types were added.

© 2021 Ravaut M et al. *JAMA Network Open.*

eFigure 3. Feature Contribution

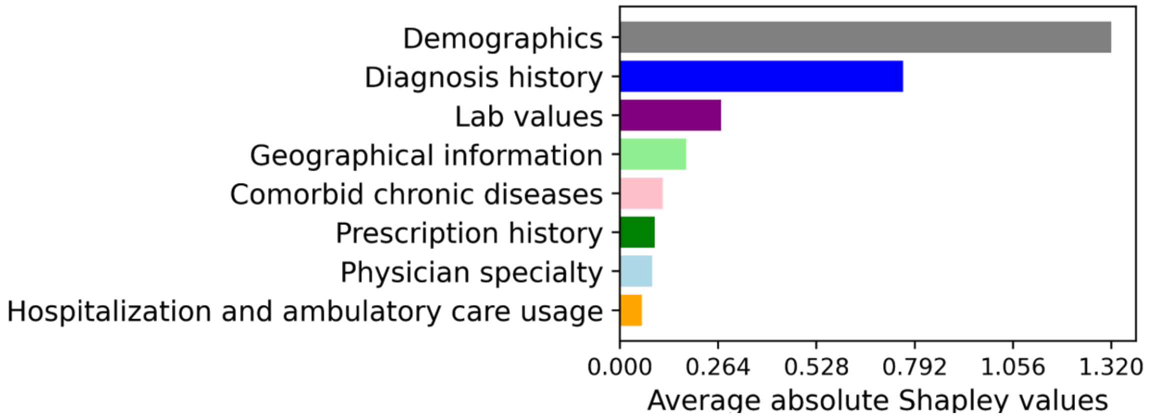

eFigure 3 displays the total contribution from each data type, computed with average absolute Shapley values. Our vastly heterogeneous input data can be organized into eight broad categories: demographics (stationary data such as country of birth or native language; but also age or landing date in Canada for immigrants), routine diagnosis codes and history, laboratory values, geographical information (including latitude and longitude), yearly flags for history of chronic diseases (i.e. asthma, hypertension, congestive heart failure, chronic obstructive pulmonary disease, Crohn's disease and arthritis), prescription history, information on the specialty of each doctor encounter, and hospitalizations.

Demographics dominate among all data categories, due to the strong contribution of age and related features such as year of birth and age at landing date in Canada for immigrants. We also note the strong contribution of diagnosis history, and moderate contribution of laboratory results. These two data types have the highest frequency among our non-stationary input data. In contrast, extreme and rarer health events such as hospitalizations or ambulatory care usage contribute less to model predictions.

© 2021 Ravaut M et al. *JAMA Network Open.*

eFigure 4. Model Precision and Recall Curves

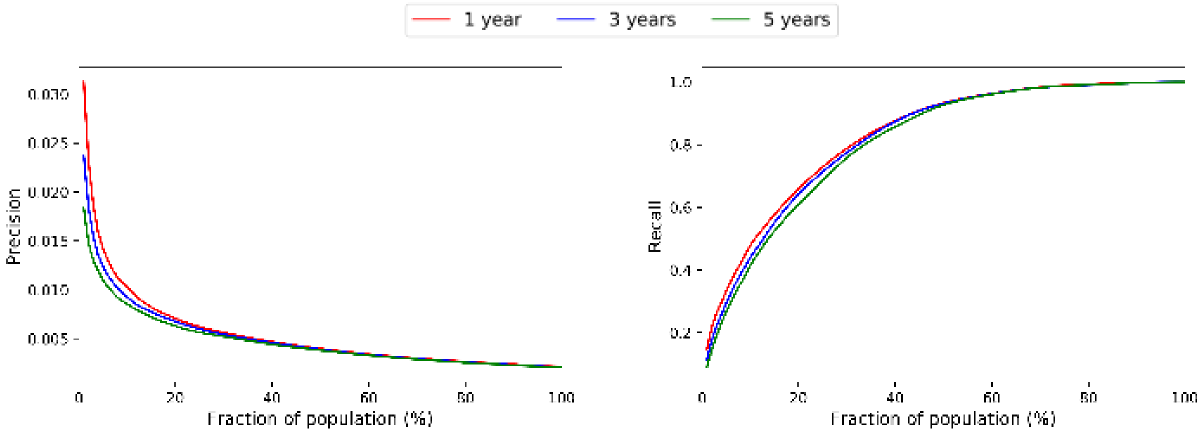

eFigure 4 shows the precision (left) and recall (right) curves for the XGBoost model for all three buffers. A shorter buffer consists in an easier prediction task and therefore leads to greater precision and recall. As we can see, most at risk patients (as predicted per the model) are more likely to develop diabetes. For instance, the recall curve indicates that the top 10% highest risk patients represent more than 40% of the actual diabetes cases.

© 2021 Ravaut M et al. *JAMA Network Open.*

eTable 3. Results With Other Buffer Sizes

Buffer size	Test AUC
1 year	82.73
3 years	81.59
5 years	80.26

We present the discriminative performance (AUC) of the XGBoost model for three different buffer values. A longer buffer means predicting diabetes onset further in advance ; which is a more difficult task.

In eTable 3, we display the test AUC achieved by a model trained in the same fashion as presented in the Results section, but with shorter buffer values of 1 year and 3 years respectively. As expected, a shorter buffer leads to a better discriminative performance.

© 2021 Ravaut M et al. *JAMA Network Open.*

eTable 4. Comparison Against Logistic Regression Baseline

**Buffer size**	**Test AUC**
1 year	79.67
3 years	78.44
5 years	77.49

We show the performance of a Logistic Regression baseline model on the test set, for the same three buffer values show in eTable 2. The input data is scaled too be within [0, 1] before training the Logistic Regression model.

In eTable 4, we display the performance of a baseline Logistic Regression model trained with the same input feature as XGBoost, for all three buffers of 1, 3, and 5 years. We preprocess each input feature to be within the range [0, 1] as we found this scaling to be crucial for Logistic Regression to reach a good performance. We tune the C regularization parameter on the validation dataset, and results presented here are with the optimal C value for each buffer. For the 5 years buffer, XGBoost performs 2.8 AUC points higher than Logistic Regression.

eReferences

1. Razavian N, Blecker S, Schmidt AM, Smith-McLallen A, Nigam S, Sontag D. Population-Level Prediction of Type 2 Diabetes From Claims Data and Analysis of Risk Factors. *Big Data*. 2015;3(4):277-287.

2. Cahn A, Shoshan A, Sagiv T, et al. Prediction of progression from pre-diabetes to diabetes: Development and validation of a machine learning model. *Diabetes Metab Res Rev*. 2020;36(2):e3252.

3. Choi BG, Rha SW, Kim SW, Kang JH, Park JY, Noh YK. Machine Learning for the Prediction of New-Onset Diabetes Mellitus during 5-Year Follow-up in Non-Diabetic Patients with Cardiovascular Risks. *Yonsei Med J*. 2019;60(2):191-199.

4. Jha AK, DesRoches CM, Campbell EG, et al. Use of electronic health records in U.S. hospitals. *N Engl J Med*. 2009;360(16):1628-1638.

5. Casanova R, Saldana S, Simpson SL, et al. Prediction of Incident Diabetes in the Jackson Heart Study Using High-Dimensional Machine Learning. *PLoS One*. 2016;11(10):e0163942.

6. Hux JE, Ivis F, Flintoft V, Bica A. Diabetes in Ontario: determination of prevalence and incidence using a validated administrative data algorithm. *Diabetes Care*. 2002;25(3):512-516.

7. Che Z, Purushotham S, Cho K, Sontag D, Liu Y. Recurrent Neural Networks for Multivariate Time Series with Missing Values. *Sci Rep*. 2018;8(1):6085.

8. Vranas KC, Jopling JK, Sweeney TE, et al. Identifying Distinct Subgroups of ICU Patients: A Machine Learning Approach. *Crit Care Med*. 2017;45(10):1607-1615.

9. Ling CX, Li C. Data mining for direct marketing: problems and solutions. In: *Proceedings of the Fourth International Conference on Knowledge Discovery and Data Mining*. KDD'98. AAAI Press; 1998:73-79.

10. Akbani R, Kwek S, Japkowicz N. Applying Support Vector Machines to Imbalanced Datasets. *Machine Learning: ECML 2004*. 2004:39-50. doi:10.1007/978-3-540-30115-8_7

11. Chawla NV, Japkowicz N, Kotcz A. Editorial: special issue on learning from imbalanced data sets. *SIGKDD Explor Newsl*. 2004;6(1):1-6.

12. Pozzolo AD, Caelen O, Johnson RA, Bontempi G. Calibrating Probability with Undersampling for Unbalanced Classification. In: *2015 IEEE Symposium Series on Computational Intelligence*. ; 2015:159-166.

13. Lundberg SM, Erion GG, Lee S-I. Consistent Individualized Feature Attribution for Tree Ensembles. *arXiv [csLG]*. February 2018. http://arxiv.org/abs/1802.03888.

14. Lundberg SM, Erion G, Chen H, et al. From local explanations to global understanding with explainable AI for trees. *Nature Machine Intelligence*. 2020;2(1):56-67.
